# S-Ketamine attenuates inflammatory effect and modulates the immune response in patients undergoing modified radical mastectomy: A prospective randomized controlled trial

**DOI:** 10.3389/fphar.2023.1128924

**Published:** 2023-02-16

**Authors:** Junxia Zhang, Qian Ma, Wenbin Li, Xiaohui Li, Xuexin Chen

**Affiliations:** ^1^ The Clinical Medical College of Ningxia Medical University, Yinchuan, Ningxia, China; ^2^ Department of Anesthesiology, Cancer Hospital, General Hospital of Ningxia Medical University, Yinchuan, Ningxia, China

**Keywords:** S-ketamine, breast cancer, immunosuppression, inflammatory factors, modified radical mastectomy

## Abstract

**Objective:** This study aimed to investigate the impact of varying dosages of S-ketamine on perioperative immune-inflammatory responses in patients undergoing modified radical mastectomy (MRM).

**Methods:** This is a prospective, randomized, controlled trial. A total of 136 patients with American Society of Anesthesiologists status I/II scheduled for MRM were enrolled and randomly assigned into groups to receive the control (C) or one of three different doses [0.25 (L-Sk), 0.5 (M-Sk), or 0.75 (H-Sk) mg/kg] of S-ketamine. The primary outcomes were the cellular immune function and inflammatory factors before anesthesia and at the end of (T1) and 24 h (T2) after the surgery. Secondary outcomes included the visual analog scale (VAS) score, opioid consumption, rate of remedial analgesia, adverse events, and patient satisfaction.

**Results:** The percentage and absolute counts of CD3^+^ and CD4^+^ cells in groups L-Sk, M-Sk, and H-Sk were higher than those of group C at T1 and T2. Moreover, a pairwise comparison revealed that the percentage in group H-Sk was higher than those in the L-Sk and M-Sk groups (*p* < 0.05). The ratio of CD4^+^/CD8^+^ was lower in group C at T1 and T2 than those in groups M-Sk and H-Sk (*p* < 0.05). There was no significant difference in the percentage and absolute counts of natural killer (NK) cells and B lymphocytes among the four groups. However, compared with group C, the concentrations of white blood cells (WBC), neutrophils (NEUT), hypersensitive C-reactive protein (hs-CRP), the neutrophil-to-lymphocyte ratio (NLR), systemic inflammation response index (SIRI), and systemic immune-inflammation index (SII) at T1 and T2 in three different doses of S-ketamine groups were significantly low, and the lymphocytes were significantly high. The ratio of SIRI and NLR at T2 in group M-Sk was lower than that in group L-Sk (*p* < 0.05). Additionally, a significant decrease in VAS score, opioid consumption, rates of remedial analgesia, and adverse events was observed in the M-Sk and H-Sk groups.

**Conclusion:** Collectively, our study demonstrates that S-ketamine could reduce opioid consumption, decrease postoperative pain intensity, exert a systemic anti-inflammatory effect, and attenuate immunosuppression in patients undergoing MRM. Moreover, we found that the effects of S-ketamine are related to the dose used, with significant differences observed in 0.5 or 0.75 mg/kg of S-ketamine.

**Clinical Trial Registration:** chictr.org.cn, identifier ChiCTR2200057226.

## 1 Introduction

The 2020 Global Cancer, Incidence, Mortality and Prevalence (GLOBOCAN) Statistics indicated that female breast cancer (BC) has officially replaced lung cancer, becoming the cancer type with the highest morbidity worldwide (Sung et al., 2021). To date, surgical resection remains the main treatment for BC. In the past few years, despite the great advancement in cancer therapy, the long-term prognosis of BC patients remains poor because of the high recurrence rate and distant metastasis. It is estimated that 30%–40% of early-stage BC patients will eventually progress to recurrence and distant metastasis (Wang et al., 2019).

Although controversial, accumulating evidence has verified that perioperative interventions, such as anesthetic agents and techniques, have been hypothesized to play a critical role in cancer recurrence or metastasis through effects on cancer cell signaling, perioperative immunomodulation, or modifying the neuroendocrine stress response (Byrne et al., 2016; Ackerman et al., 2021). Emerging evidence supports that anesthesia and related drugs can directly or indirectly affect the immune system and increase the likelihood of cancer dissemination and metastasis (Connolly and Buggy, 2016; Kim, 2017). Accordingly, anesthetic agents should be scientifically selected to attenuate perioperative immunosuppression, which is critical to prevent BC recurrence and metastasis and improve the long-term prognosis of BC patients.

S-Ketamine is an isomer of ketamine, a non-selective N-methyl-D-aspartate (NMDA) receptor antagonist (Aronsohn et al., 2019). Growing evidence suggests that ketamine has antidepressant, anti-inflammatory (Nowak et al., 2019), and antitumor effects (Zhou et al., 2018). It also proposed that ketamine showed protective effects against cellular immune impairment and cancer metastasis induced by surgical stress (Forget et al., 2010). Furthermore, clinical research also found that low-dose S-ketamine can reduce early postoperative serum proinflammatory cytokine (IL-6) levels in elective surgical patients (Luggya et al., 2017). However, research on S-ketamine mainly focuses on antidepressant therapy, and little is known about the effect of S-ketamine on immune-inflammatory response in BC patients, which may be a potential agent to attenuate immunosuppression in cancer patients.

Therefore, this study aimed to evaluate the effect of different doses of S-ketamine on cellular immune function and inflammatory factors in patients undergoing modified radical mastectomy (MRM). Based on previous studies, our primary hypothesis was that the S-ketamine administration during the perioperative period could not only reduce opioid consumption but also play an anti-inflammatory and immune protective role.

## 2 Methods

### 2.1 Patients

This study was approved by the Ethics Committee of The General Hospital of Ningxia Medical University (no. KYLL-2022-0056) and registered at http://www.chictr.org.cn/index.aspx (no. ChiCTR2200057226). After obtaining written informed consent, female patients aged 30–75 years with American Society of Anesthesiologists (ASA) physical status I/II who were scheduled for MRM were enrolled. Patients with a history of severe heart, lung, liver, kidney, or endocrine diseases, history of allergy to the drug being studied, cognitive impairment, use of antipsychotics, or use of non-steroidal anti-inflammatory drugs (NSAIDs) were excluded.

Patients were randomly assigned to one of four treatment groups (control, low-dose, medium-dose, or high-dose S-ketamine) following a 1:1:1:1 ratio using a computer-generated random number table. The group allocation was concealed in sealed and opaque envelopes, and the corresponding envelopes were only opened when the patient was allocated to the intervention. The study medications were prepared by an attending anesthetist so that the designated researcher was blinded to the intervention. Group L-Sk received S-ketamine 0.25 mg/kg for induction, followed by an infusion of 0.25 mg/kg/h. Group M-Sk received S-ketamine 0.5 mg/kg for induction, followed by an infusion of 0.5 mg/kg/h. Group H-Sk received S-ketamine 0.75 mg/kg for induction, followed by an infusion of 0.75 mg/kg/h. Group C did not receive S-ketamine during anesthesia. Lavender suggested that an induction dose of S-ketamine is 0.5–1 mg/kg, and the maintenance dose is 0.5–3 mg/kg (Lavender et al., 2020). The S-ketamine dose in this study was within a safe dose range.

### 2.2 Anesthesia and perioperative care

All patients were intramuscularly injected with 1 mg of penehyclidine 30 min before surgery. Routine monitoring includes an electrocardiography, pulse oximetry, heart rate, non-invasive blood pressure, and bispectral index. The anesthesia induction and maintenance methods among the four groups were as follows: patients in group C were induced with midazolam (0.05 mg/kg), sufentanil (0.3 μg/kg), propofol (2.0 mg/kg), and rocuronium (0.8 mg/kg) and maintained with a target-controlled infusion of propofol (4–6 mg/kg/h) and remifentanil (0.2–0.6 ug/kg/min). Patients in three different doses of the S-ketamine group were induced with midazolam (0.05 mg/kg), S-ketamine (0.25, 0.5, or 0.75 mg/kg), propofol (2.0 mg/kg), and rocuronium (0.8 mg/kg) and maintained with a target-controlled infusion of propofol (4–6 mg/kg/h), remifentanil (0.2–0.6 ug/kg/min), and S-ketamine (0.25, 0.5, or 0.75 mg/kg/h).

The anesthetic administration rate was adjusted to the study protocol’s maintenance dose to maintain a bispectral index range of 40–60 and mean arterial pressure (MAP) within 20% of preoperative baseline values. Ephedrine or norepinephrine was administered if the patient had hypotension (defined as a decrease in arterial blood pressure of more than 20% of the basic value). Urapidil, alpha 1 antagonist, was administered if the patient had hypertension (defined as an increase in arterial blood pressure of more than 20% of the basic value). S-Ketamine infusion stopped 30 min before the end of surgery. Drugs possessing anti-inflammatory effects, such as dexamethasone, etomidate, lidocaine, and dexmedetomidine, were not administered during the perioperative period in all patients. Patients were transferred to the post-anesthesia care unit (PACU) for recovery.

### 2.3 Outcomes

The primary outcomes were cellular immune function and systemic inflammatory markers. A volume of 4 ml of peripheral blood from the elbow of all patients was collected before anesthesia (T0) and at the end of (T1) and 24 h after the surgery (T2). The percentage of T lymphocytes (CD3^+^, CD4^+^, and CD8^+^), B lymphocytes, and natural killer (NK) cells, the ratio of CD4^+^/CD8^+^, and the counts of white blood cells (WBC), neutrophils (NEUT), lymphocytes (LYM), and hypersensitive C-reactive protein (hs-CRP) were compared between the groups. The inflammation-based prognostic scores were calculated, such as neutrophil-to-lymphocyte ratio (NLR), lymphocyte-to-monocyte ratio (LMR), platelets-to-lymphocyte ratio (PLR), systemic inflammation response index (SIRI), and systemic immune-inflammation index (SII). The SIRI and SII were calculated using the following formulas: SIRI = neutrophil count × monocyte count/lymphocyte count and SII = platelet count × neutrophil count/lymphocyte count. Lymphocyte subset analysis and lymphocyte percentage were detected by flow cytometry (FCM) to reflect the cellular immune function.

The secondary outcome included postoperative pain intensity, cumulative opioid and propofol consumption, PACU recovery time, awakening time (it refers to the time from drug withdrawal to extubation), adverse events (e.g., the occurrence of shivering, nausea, vomiting, dizziness, agitation, hypertension, psychotomimetic side effects—hallucinations or nightmares—and other adverse reactions), the incidence of remedial analgesia, and patient satisfaction score. Postoperative pain intensity was evaluated using an 11-point visual analogue scale (VAS) score at the end of the surgery and 4, 6, 24, and 48 h after the surgery. A straight line was divided into nine equal parts, and the two ends represented 0 points of no pain and 10 points of severe pain. The patients used a line to mark the value according to their subjective feelings to reflect the pain degree (Dannenbaum et al., 2011). If the postoperative pain exceeded 3 points, an intramuscular injection of diclofenac sodium of 75 mg was carried out for remedial analgesia. If the patient had moderate-to-severe nausea or vomiting, a serotonin 5-HT3 antagonist (tropisetron) of 2 mg was given intravenously. Patient satisfaction was assessed 48 h postoperatively by patients using an 11-point Likert scale (range, 0–10), where a score of 0 means “entirely unsatisfied,” and a score of 10 means “fully satisfied” (Farooq et al., 2016).

### 2.4 Statistical analysis

Statistical analysis was performed using SPSS 27.0. The Kolmogorov–Smirnov test and the Q–Q plot were used to assess the normality of all data. Normally distributed variables were analyzed using one-way analysis of variance (ANOVA). A Bonferroni *post hoc* test was used to compare groups pairwise. Continuous variables not following a normal distribution were analyzed using the Kruskal–Wallis test with a Dunn *post hoc* test used to compare groups pairwise. The inflammation index and ratio of lymphocyte subsets were compared using ANOVA for repeated measures. Enumerated data were presented as a percentage or ratio, and the chi-square test was used for comparisons. *p* < 0.05 was considered significant.

Based on our preliminary data (*n* = 30, 15 cases in group C, 15 cases in group M-Sk), the percentage of CD3^+^ in group C at the end of surgery was (55.51 ± 5.67), that in group M-Sk was (60.25 ± 4.48), the sample size required to detect a significant difference in immune function was calculated with an online program (http://powerandsamplesize.com/Calculators/Compare-k-Means/1-Way-ANOVA-Pairwise), and the results showed that 25 subjects in each group are needed, ensuring that the power of the test is 80% with a type alpha error of 0.05 (the alpha error was corrected by six pairwise comparisons). Allowing for dropouts, we aimed to recruit 35 patients in each group, with a total of 140 patients.

## 3 Results

### 3.1 Demographic and perioperative characteristics of enrolled participants

**FIGURE 1:**
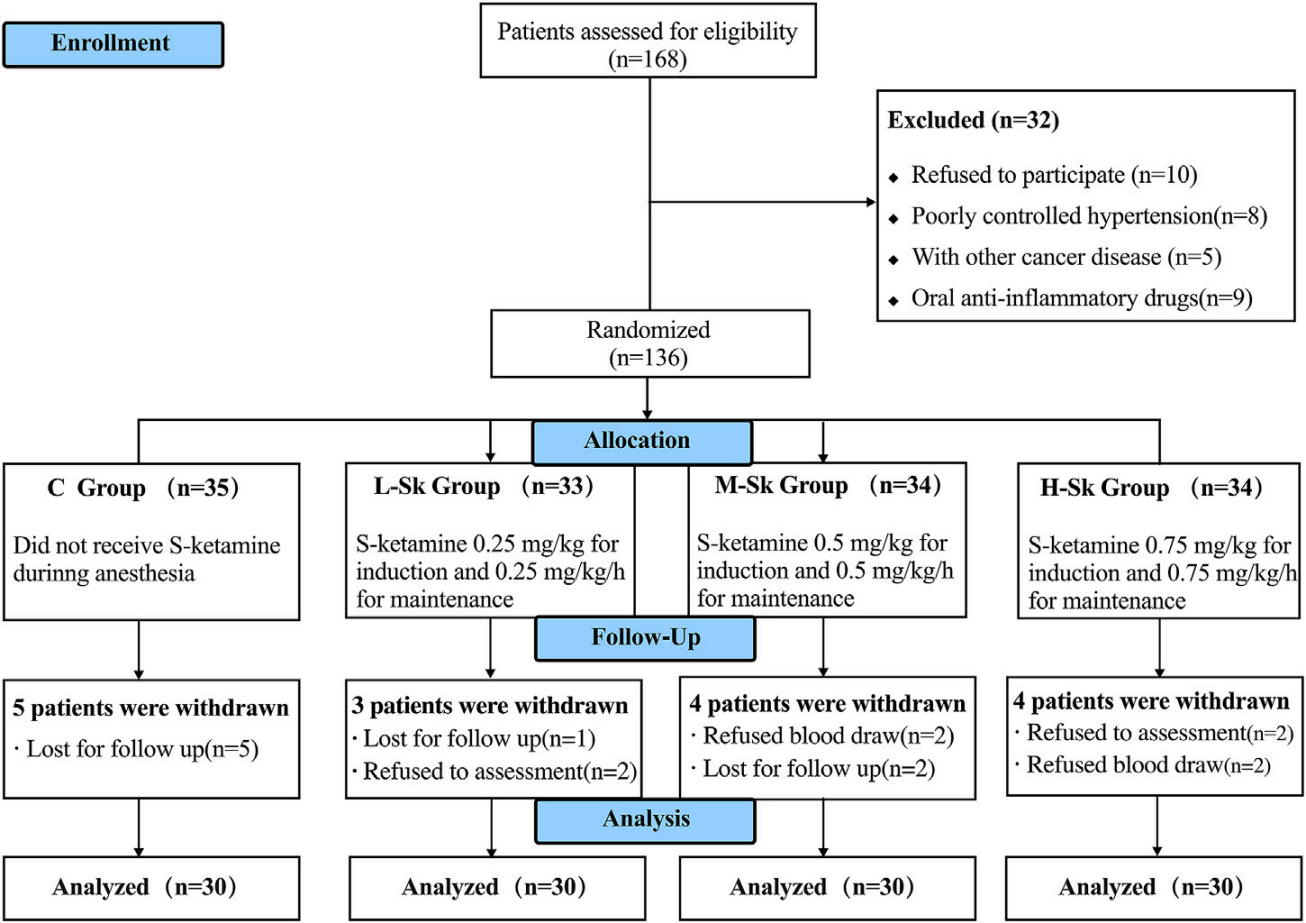
Flowchart of Consolidated Standards of Reporting Trials (CONSORT) describing patients’ progress throughout the study.

A total of 168 patients were first assessed for eligibility in this study. However, 32 patients met the exclusion criteria. Thus, 136 patients were randomized and allocated to the study groups. Later, five participants in group C, three in group L-Sk, four in group M-Sk, and four in group H-Sk were excluded due to loss to follow-up and refusing postoperative assessment or postoperative blood draw. Consequently, 120 patients were analyzed, with 30 patients in each group (Figure 1).

The baseline characteristics and perioperative data are presented in Table 1. There were no significant differences with respect to patient demographic data, the duration of anesthesia and surgery, PACU recovery time, and awakening time among the four groups (*p* > 0.05). Because of the analgesic effects of S-ketamine, three different doses of S-ketamine (0.25, 0.5, and 0.75 mg/kg) effectively reduced the consumption of opioids compared with the C group (F = 62.45, *p* < 0.001). Further pairwise comparison indicated that the consumption of opioids was significantly decreased in the M-Sk and H-Sk groups compared to the C and L-Sk groups (Bonferroni correction, *p* < 0.001). However, a non-significant reduction was observed between the H-Sk and M-Sk groups. Additionally, according to the experimental design principles, the different doses of the S-ketamine group did not use sufentanil (*p* < 0.001). These results suggested that the use of S-ketamine can effectively reduce opioid consumption, especially in 0.5 or 0.75 mg/kg (Table 1).

**TABLE 1:** Demographic data and perioperative characteristics.

Variable	C group (*n* = 30)	L-Sk group (*n* = 30)	M-Sk group (*n* = 30)	H-Sk group (*n* = 30)	*p*-value
Age (years)	56.0 ± 8.2	53.7 ± 11.6	52.3 ± 11.3	50.7 ± 7.2	0.19
BMI (kg/m^2^)	21.8 ± 2.0	22.4 ± 2.3	22.6 ± 1.7	22.7 ± 2.4	0.28
ASA (I/II)	8/22	10/20	10/20	7/23	0.82
Menopause (%)	24 (80%)	21 (70%)	20 (66.7%)	21 (70%)	0.68
Duration of anesthesia (min)	117.0 ± 26.6	107.9 ± 20.2	108.4 ± 15.7	107.4 ± 15.8	0.21
Duration of surgery (min)	100.1 ± 25.8	90.1 ± 19.7	89.0 ± 18.4	91.5 ± 16.2	0.15
S-Ketamine (mg)	0.0	26.2 ± 4.6*	52.1 ± 10.0*, ^#^	70.2 ± 12.7*, ^#^	<0.001
Propofol (mg)	330.0 ± 79.0	253.0 ± 67.7*	255.0 ± 65.4*	207 ± 40.3*, ^#^	<0.001
PACU recovery time	38.4 ± 8.0	36.3 ± 8.3	33.3 ± 6.1	37.2 ± 10.1	0.11
Awakening time (min)	7.0 ± 1.5	6.5 ± 1.4	6.4 ± 1.4	6.7 ± 1.3	0.35
Opioid consumption Remifentanil (mg)	1.7 ± 0.3	1.1 ± 0.3*	0.9 ± 0.2*, ^#^	0.7 ± 0.2*, ^#^	<0.001
Sufentanil (ug)	18.3 ± 1.6	0.0*	0.0*	0.0*	<0.001

Values are presented as mean ± SD and analyzed using one-way analysis of variance (ANOVA). The Bonferroni *post hoc* test was used to compare groups pairwise. Values are presented as the number of patients (%) and analyzed using the chi-squared test or Fisher exact test as appropriate. If the overall test of difference among groups was significant, chi-squared tests were used for pairwise comparisons. BMI, body mass index; ASA, American Society of Anesthesiologists; PACU, post-anesthesia care unit; C group, control group; L-Sk group, low-dosage S-ketamine group; M-Sk group, middle-dosage S-ketamine group; H-Sk group, high-dosage S-ketamine group.

*p < 0.05 compared to group C.

^#^ *p* < 0.05 compared to group L-Sk.

^△^ *p* < 0.05 compared to group M-Sk.

### 3.2 Comparison of the cellular immune function among the four groups

**FIGURE 2:**
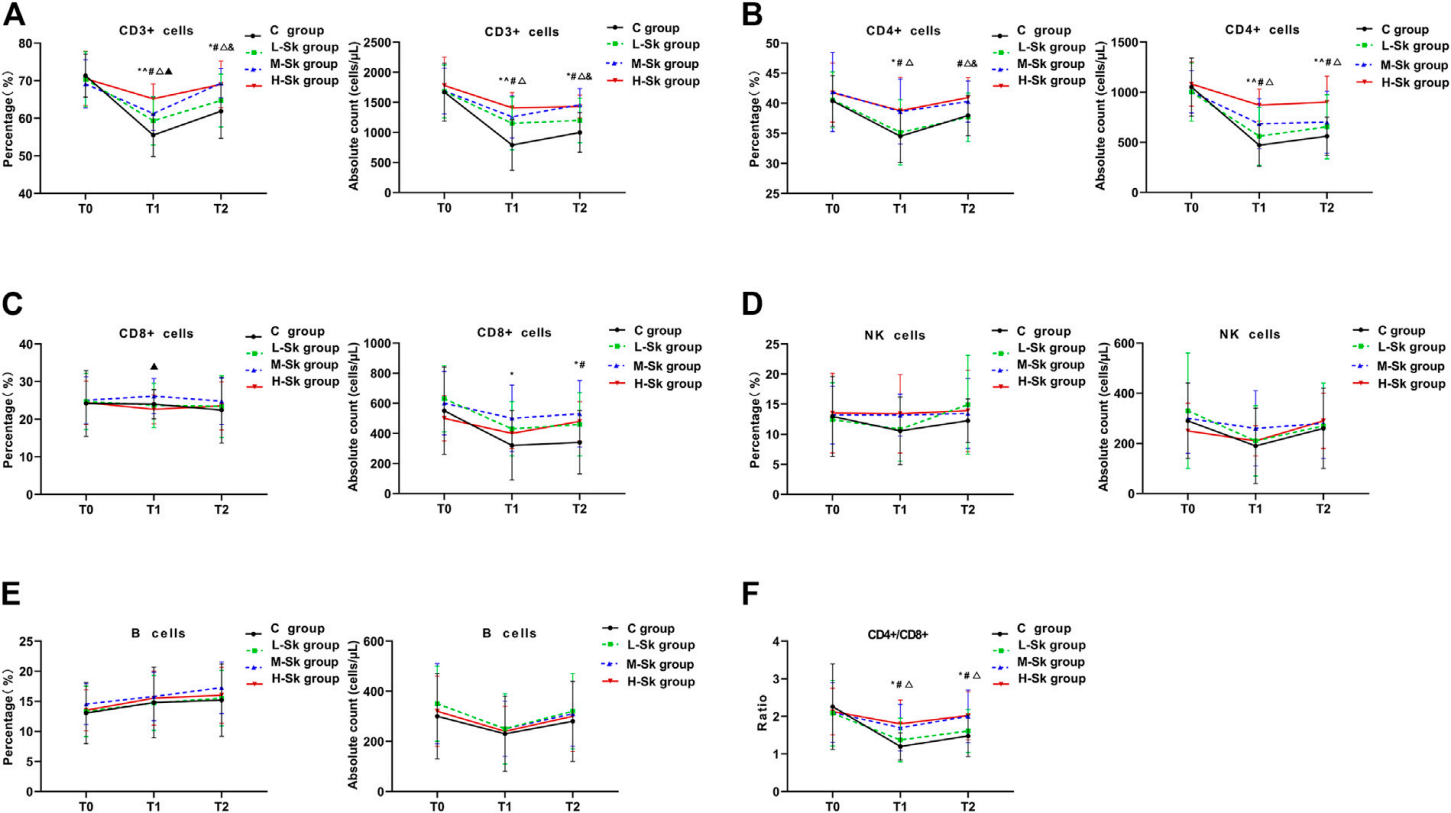
Comparison of the percentage and absolute counts of the lymphocyte subsets among the four groups. The cellular immune function variables include CD3^+^ cells **(A)**, CD4^+^ cells **(B)**, CD8^+^ cells **(C)**, NK cells **(D)**, B cells **(E)**, and CD4^+^/CD8^+^ **(F)**. Values represent the estimated means from linear mixed models with the standard error, analyzed using ANOVA for repeated measures. T0, before anesthesia; T1, at the end of surgery; T2, 24 h after the surgery; C group, control group; L-Sk group, low-dosage S-ketamine group; M-Sk group, middle-dosage S-ketamine group; H-Sk group, high-dosage S-ketamine group. ^*p* < 0.05, the L-Sk *vs* C groups at the corresponding time point. *p < 0.05, the M-Sk *vs* C groups at the corresponding time point. ^#^ *p* < 0.05, the H-Sk *vs* C groups at the corresponding time point. ^△^ *p* < 0.05, the H-Sk *vs* L-Sk groups at the corresponding time point. ^&^ *p* < 0.05, the M-Sk *vs* L-Sk groups at the corresponding time point. ^▲^ *p* < 0.05, the H-Sk *vs* M-Sk groups at the corresponding time point.

The percentage and absolute counts of the lymphocyte subsets detected by FCM among the four groups are shown in Figure 2. Existing studies have verified that the low counts of CD3^+^, CD4^+^, and NK cells are associated with impaired antitumor immunity. When the ratio of CD4+/CD8+ decreases, the immune balance is damaged.

In this study, the preoperative cellular immune function was comparable among the four groups. However, at the end of the surgery, the percentage of CD3^+^ cells in the C group was significantly lower than that in the S-ketamine groups. Moreover, a pairwise comparison indicated that the percentage of CD3^+^ cells in group H-Sk was higher than the percentage in groups L-Sk and M-Sk (*p* < 0.05). Similarly, 24 h after the surgery, the percentage of CD3^+^ cells in groups M-Sk and H-Sk were higher than those in groups C and L-Sk (*p* < 0.05) (Figure 2A). The percentage of CD4^+^ cells at T1 in group C was lower than the percentage in groups M-Sk and H-Sk, and the percentages were higher in group H-Sk than that in group L-Sk (*p* < 0.05). However, at T2, the percentage of CD4^+^ in group C was lower than that in group H-Sk. Groups H-Sk and M-Sk had a higher percentage of CD4^+^ than group L-Sk (*p* < 0.05) (Figure 2B). There was a lower percentage of CD8^+^ cells at T1 in group H-Sk than in group M-Sk (*p* < 0.05) (Figure 2C). As for the lymphocyte subset absolute counts, the absolute counts of CD3^+^, CD4^+^, and CD8^+^ cells in the C group were significantly lower than those in the S-ketamine groups (*p* < 0.05). A pairwise comparison indicated that the absolute counts of CD3^+^ and CD4^+^ cells in group H-Sk were higher than those in group L-Sk (*p* < 0.05). The ratios of CD4^+^/CD8^+^ at T1 and T2 in group C were lower than those in groups H-Sk and M-Sk. Furthermore, the ratio in group H-Sk was higher than that in group L-Sk (*p* < 0.05) (Figure 2F). Additionally, there was no significant difference in the percentage and absolute counts of NK cells and B lymphocytes among the four groups (Figures 2D, E). These results indicated that S-ketamine is closely related to perioperative immune protection, and the protective effect in the M-Sk and H-Sk groups may be more obvious (Figure 2).

### 3.3 Comparison of the systemic inflammatory responses among the four groups

**FIGURE 3:**
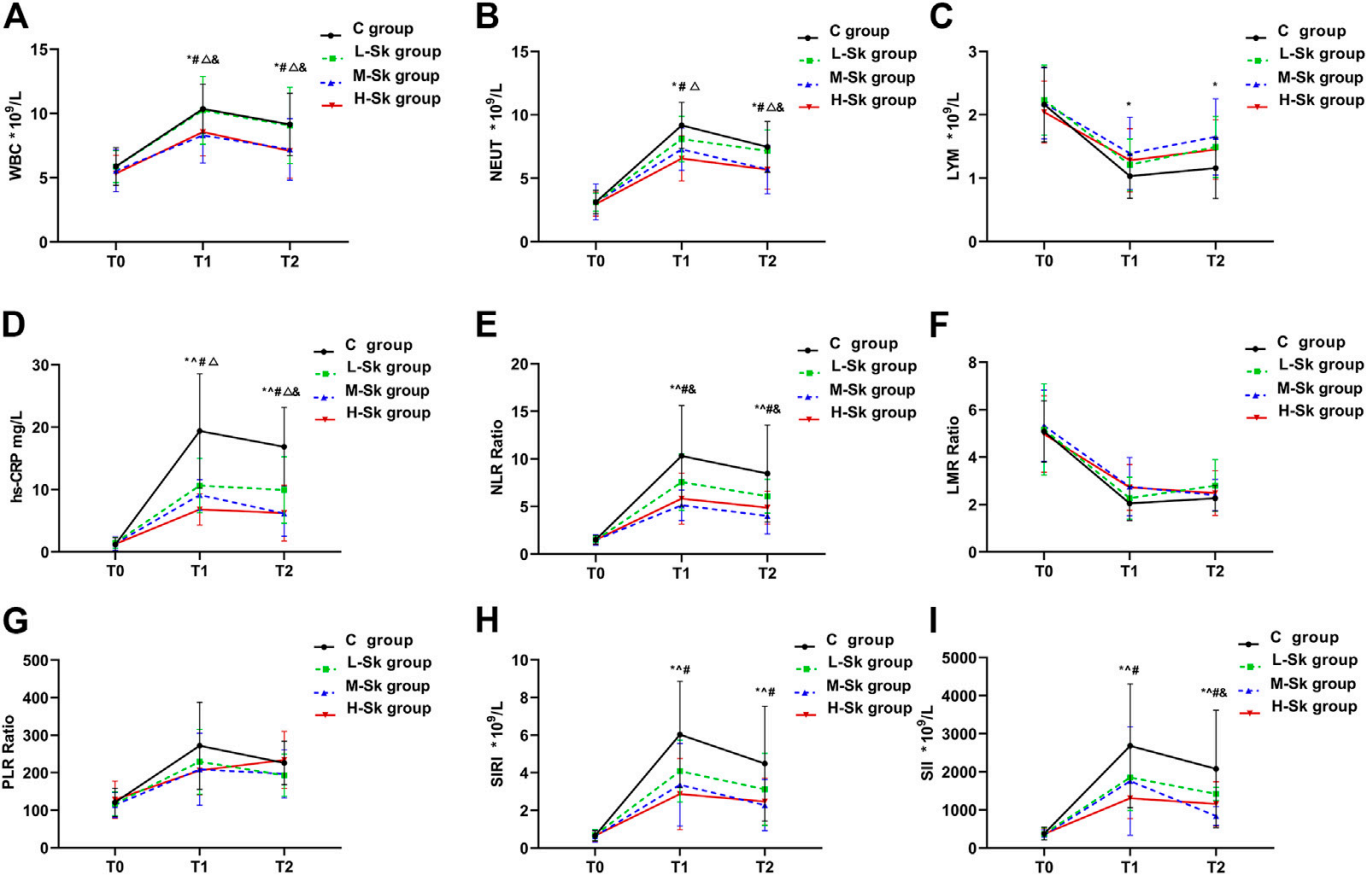
Comparison of the systemic inflammatory responses among the four groups. The system inflammatory variables include WBC **(A)**, NEUT **(B)**, LYM **(C)**, hs-CRP **(D)**, NLR **(E)**, LMR **(F)**, PLR **(G)**, SIRI **(H)**, and SII **(I)**. Values represent the estimated means from linear mixed models with the standard error, analyzed using ANOVA for repeated measures. T0, before anesthesia; T1, at the end of the surgery; T2, 24 h after the surgery; C group, control group; L-Sk group, low-dosage S-ketamine group; M-Sk group, middle-dosage S-ketamine group; H-Sk group, high-dosage S-ketamine group. WBC, white blood cells; NEUT, neutrophils; LYM, lymphocytes; NLR, neutrophil-to-lymphocyte ratio; LMR, lymphocyte-to-monocyte ratio; PLR, platelets-to-lymphocyte ratio; SIRI, systemic inflammation response index; SII, systemic immune-inflammation index. ^*p* < 0.05, the L-Sk *vs* C groups at the corresponding time point. *p < 0.05, the M-Sk *vs* C groups at the corresponding time point. ^#^ *p* < 0.05, the H-Sk *vs* C groups at the corresponding time point. ^△^ *p* < 0.05, the H-Sk *vs* L-Sk groups at the corresponding time point. ^&^ *p* < 0.05, the M-Sk *vs* L-Sk groups at the corresponding time point. ^▲^ *p* < 0.05, the H-Sk *vs* M-Sk groups at the corresponding time point.

The systemic inflammatory responses among the four groups are depicted in Figure 3. Studies have indicated that inflammation plays a vital role in tumorigenesis and progression. Mounting evidence suggests that high levels of WBC, NEUT, hs-CRP, NLR, PLR, SII, and SIRI are positively associated with poor overall survival and disease-free survival and negatively associated with LMR in patients with cancer.

The results of the present study showed that the preoperative inflammatory responses were comparable among the four groups. As shown in Figures 3A, B, at T1 and T2, compared with the C group, the concentrations of WBC and NEUT were significantly decreased in the M-Sk and H-Sk groups (*p* < 0.05). Furthermore, the pairwise comparison indicated that the concentrations of WBC and NEUT in group H-Sk were higher than that in group L-Sk (F = 7.589, *p* = 0.0001 < 0.05). Compared with group C, the concentration of LYM at T1 and T2 in group M-Sk was significantly increased (*p* < 0.05) (Figure 3C). The concentration of hs-CRP and the ratio of NLR at T1 and T2 in groups L-Sk, M-Sk, and H-Sk were significantly decreased compared to group C. The ratio of NLR in group M-Sk was lower than that in group L-Sk. A lower concentration of hs-CRP was observed in group H-Sk compared to group L-Sk. Furthermore, the concentration of hs-CRP at T2 in group L-Sk was higher than that in group M-Sk (*p* < 0.05) (Figures 3D, E). The ratios of SIRI and SII both at T1 and T2 in groups L-Sk, M-Sk, and H-Sk were significantly decreased compared to group C, and the ratio of SII at T2 in group M-Sk was lower than that in group L-Sk (*p* < 0.05) (Figures 3H, I). There were no significant differences in terms of the ratios of LMR and PLR at the corresponding time point among the four groups (*p* > 0.05) (Figures 3F, G). The aforementioned results indicate that S-ketamine might have anti-inflammatory effects. Moreover, the experimental data showed that the anti-inflammatory effects in groups M-Sk and H-Sk may be more significant (Figure 3).

### 3.4 Comparison of the perioperative adverse reactions and patient satisfaction in all groups

The perioperative adverse reactions and patient satisfaction are presented in Table 2. The incidence of postoperative nausea and vomiting (PONV) in group C was 10 cases (33.3%), which was higher than that in the groups L-Sk, M-Sk, and H-Sk (*p* < 0.05), and non-difference was observed among the S-ketamine groups. The incidence of shivering in groups M-Sk and H-Sk was 0 cases (0%), which was lower than that in group C (16.7%) (*p* < 0.05) (Table 2). There were no significant differences in terms of dizziness, agitation, hypertension, and other adverse reactions among the four groups, and no patient exhibited psychotomimetic side effects related to S-ketamine. S-ketamine has a slight excitatory effect on the circulatory system, often causing a slight increase in blood pressure and heart rate. Therefore, our study has shown that the rates of hypotension and using vasoactive drugs in groups L-Sk, M-Sk, and H-Sk were lower than those in group C, and the rates were lower in group H-Sk than those in group L-Sk (*p* < 0.001). Furthermore, compared with group C, the anesthesia satisfaction score in groups M-Sk and H-Sk increased significantly and was higher in group M-Sk than in group L-Sk (*p* < 0.001). Similarly, the rate of remedial analgesia in group C was eight cases (26.7%), which was higher than that in groups M-Sk (3.3%) and H-Sk (6.7%) (*p* < 0.05). These results suggest that S-ketamine can be used safely and effectively in cancer patients without increasing the incidence of complications (Table 2).

**TABLE 2:** Perioperative adverse reactions and patient’s satisfaction score.

Variable	C group (*n* = 30)	L-Sk group (*n* = 30)	M-Sk group (*n* = 30)	H-Sk group (*n* = 30)	*p*-value
PONV	10 (33.3%)	2 (6.7%)*	2 (6.7%)*	3 (10.0%)*	0.006
Dizziness	3 (10.0%)	2 (6.7%)	3 (10.0%)	5 (16.7%)	0.61
Agitation	2 (6.7%)	2 (6.7%)	2 (6.7%)	4 (13.3%)	0.77
Shivering	5 (16.7%)	2 (6.7%)	0 (0.0%)*	0 (0.0%)*	0.01
Hypotension	13 (43.3%)	5 (16.7%)*	2 (6.7%)*	0 (0.0%)*^, #^	<0.001
Hypertension	3 (10.0%)	3 (10.0%)	1 (3.3%)	5 (16.7%)	0.39
Ephedrine	11 (36.7%)	3 (10.0%) *	1 (3.3%)*	0 (0.0%)*	<0.001
Urapidil	3 (10.0%)	2 (6.7%)	1 (3.3%)	3 (10.0%)	0.72
Tropisetron	6 (20.0%)	2 (6.7%)	1 (3.3%)	1 (3.3%)	0.06
Diclofenac	8 (26.7%)	3 (10.0%)	1 (3.3%)*	2 (6.7%)*	0.02
Patient satisfaction	8.0 ± 1.4	8.5 ± 1.3	9.3 ± 0.8*^, #^	9.0 ± 0.7*	<0.001

Values are presented as mean ± SD and analyzed using one-way analysis of variance (ANOVA). The Bonferroni *post hoc* test was used to compare groups pairwise. Values are presented as the number of patients (%) and analyzed using the chi-squared test or Fisher exact test as appropriate. If the overall test of difference among groups was significant, chi-squared tests were used for pairwise comparisons. PONV, postoperative nausea, and vomiting; C group, control group; L-Sk group, low-dosage S-ketamine group; M-Sk group, middle-dosage S-ketamine group; H-Sk group, high-dosage S-ketamine group.

*p < 0.05 compared to group C.

^#^ *p* < 0.05 compared to group L-Sk.

^△^ *p* < 0.05 compared to group M-Sk.

### 3.5 Comparison of the VAS scores of patients in all groups

**FIGURE 4:**
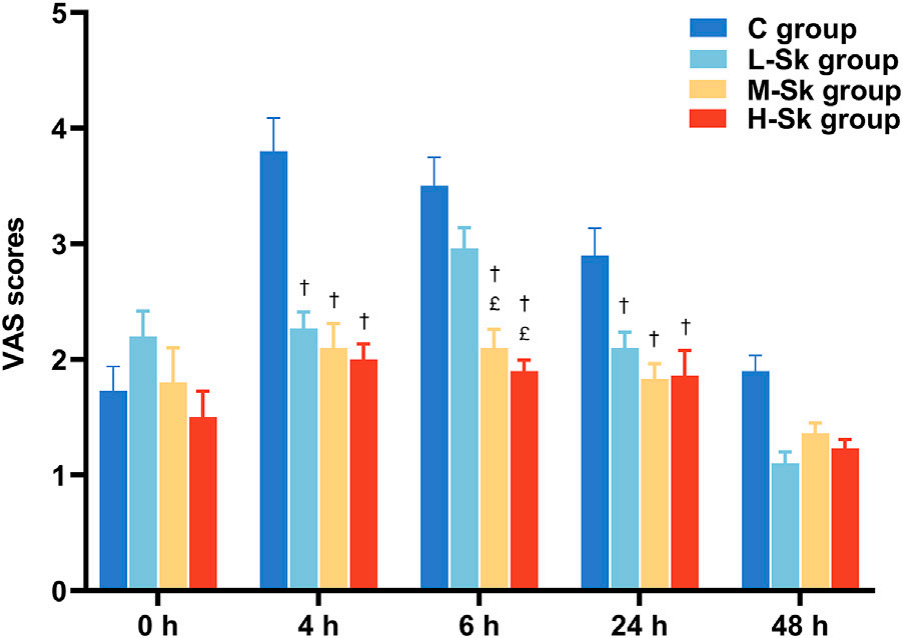
Comparison of the VAS scores for patients in all groups. 0 h, the end of the surgery; 4 h, 4 h after the surgery; 6 h, 6 h after the surgery; 24 h, 24 h after the surgery; 48 h, 48 h after the surgery; C group, control group; L-Sk group, low-dosage S-ketamine group; M-Sk group, middle-dosage S-ketamine group; H-Sk group, high-dosage S-ketamine group. ^†^ *p* < 0.05 compared to the C group. ^£^ *p* < 0.05 compared to the L-Sk group. ^‡^ *p* < 0.05 compared to the M-Sk group.

Postoperative VAS scores for participants are illustrated in Figure 4. Briefly, no statistically significant differences in pain scores were detected among the four groups at the end of and 48 h after the surgery. However, 4 and 24 h after the surgery, the VAS scores in the L-Sk, M-Sk, and H-Sk groups decreased significantly compared to the C group. There were lower VAS scores at 6 h in the M-Sk and H-Sk groups than in the C and L-Sk groups (*p* < 0.05). However, no significant difference was detected between the M-Sk and H-Sk groups. These results suggest that the S-ketamine doses of 0.5 or 0.75 mg/kg showed a better analgesic effect; it can reduce the intensity of postoperative pain, which means S-ketamine is a promising option for perioperative analgesia (Figure 4).

## 4 Discussion

This trial estimated the effect of three different doses (0.25, 0.5, and 0.75 mg/kg) of S-ketamine on immune-inflammatory function in patients undergoing MRM. According to the results obtained from this study, the S-ketamine doses of 0.5 or 0.75 mg/kg have less impact on patients’ cell-mediated immunity function, and the inflammation response is lighter. Additionally, we found beneficial effects of S-ketamine on postoperative pain, opioid consumption, and anesthesia satisfaction.

It is critical to elucidate the relationship between anesthetics and immune function because the immune system plays an essential role in facilitating or hindering tumor growth and metastasis (Wall et al., 2019). T lymphocytes participate in the immune system and are extensively involved in immune regulation, inflammation, and protective immune mechanisms. CD3^+^, CD4^+^, and CD8^+^ T cells are the main T lymphocyte cells in antitumor immunity (Tu et al., 2021). Existing studies have suggested that patients with low T lymphocytes, CD4+/CD8+ ratio, and NK cell activity are predisposed to worse outcomes (O'Dwyer et al., 2015).

In our study, the total percentage of T lymphocytes was significantly decreased after MRM, especially in the control group. However, the application of S-ketamine can attenuate postoperative immunosuppression, mainly manifested by the increased percentages of CD3^+^ and CD4^+^ T cells and the higher ratios of CD4+/CD8+ at different time points in the S-ketamine groups. Our results are in accordance with several previous studies. Forget et al. (2010) verified that ketamine showed protective effects against cellular immune impairment induced by surgical stress. Furthermore, the results of Wang et al. (2021) confirmed that 0.5 mg/kg of S-ketamine can alleviate the inhibitory effect of postoperative cellular immune function in patients undergoing gynecological laparoscopic surgery. However, contradictory results have been reported on whether ketamine has an anti-immunosuppression effect. Cho et al. (2021) believed that intraoperative low-dose ketamine administration did not convey any favorable impacts on overall postoperative NK cell activity, inflammatory responses, and prognosis in colorectal cancer surgery patients. These results may be partly attributable to relatively less postoperative inflammatory activation in laparoscopic surgery and low-dose ketamine use. Therefore, multiple clinical trials need to be conducted to verify this conclusion.

We have evidence that S-ketamine produced an anti-inflammatory effect by mitigating excessive systemic inflammation indicators’ release, such as the expression of WBC, NEUT, hs-CRP, NLR, SII, and SIRI, as well as reducing the inhibition of LYM. Moreover, S-ketamine doses of 0.5 and 0.75 mg/kg showed better anti-inflammatory effects compared to the 0.25 mg/kg dose. In addition, previous studies have confirmed that NLR, LMR, SIRI, and SII are associated with cancer recurrence and poor prognosis in many types of cancer (Moon et al., 2020; Kinoshita and Goto, 2021), including BC, endometrial cancer, gastric carcinoma, and colon cancer. Therefore, based on previous studies and our results, we hypothesized that the use of S-ketamine may have a beneficial effect on the prognosis of patients with cancer. These results are consistent with the trend of previous studies. For example, Wang et al. (2022) reported that S-ketamine alleviates postoperative depression-like behavior through anti-inflammatory actions. Tu et al. (2021) demonstrated that S-ketamine has anti-inflammatory effects, can inhibit the release of inflammatory cells, and can reduce the secretion of interleukin, tumor necrosis factor, and other cytokines by leucocytes, thus alleviating the inflammatory response. In contrast, an anti-inflammatory effect of ketamine was not observed by Cho et al. (2021). This could be linked to less postoperative inflammatory activation in laparoscopic surgery. Our results confirmed that S-ketamine plays an immune-protective role by decreasing the systemic production of inflammatory factors induced by surgery and attenuating immunosuppression in patients undergoing breast surgery.

Furthermore, most studies have shown that opioids may impair the perioperative immune system and increase vascular permeability, which may promote tumor cell growth (Byrne et al., 2016; Ben-David, 2016). Bimonte et al. (2015) found that opioid-sparing analgesia may help keep the function of NK cells and reduce the metastatic spread of cancer. Likewise, our results demonstrated that perioperative S-ketamine administration was effective in assisting analgesia, which can significantly decrease the consumption of remifentanil and sufentanil. Postoperative pain could lead to the release of inflammatory factors, resulting in immune disorder (Ao et al., 2021). In this study, the postoperative VAS scores of the patients in group C showed a higher score, indicating that S-ketamine provided better postoperative pain control by decreasing the intensity of postoperative pain and the requirement of additional remedial analgesia. Satisfactory postoperative pain management is crucial to assuring good patient experience and may potentially decrease the risk of immune disorder. These results are consistent with previous research studies (Nielsen et al., 2019; Ithnin et al., 2019). In addition, we confirmed that S-ketamine in 0.5 and 0.75 mg/kg doses had significant effects on opioid-sparing and reduced the VAS scores and the rate of remedial analgesia. We assumed that the use of S-ketamine anesthesia may indirectly prevent perioperative immunosuppression during surgery because it can significantly decrease opioid consumption and the probability of remedial analgesia. In other words, if the immunosuppression brought by opioids could be abolished by S-ketamine, it would increase the available choices of anesthetic methods for cancer patients and may suppress the postoperative incidence of metastasis and improve patients’ long-term survival.

Regarding the safety profile of S-ketamine in our study, none of the patients presented any other complaints attributable to S-ketamine. Although we observed that with the increase in the dose of S-ketamine, the incidence of dizziness and agitation increased gradually, there was no statistical significance. We also found that S-ketamine can reduce the incidence of PONV, consistent with Brinck’s research (Brinck et al., 2018). Moreover, Zhang et al. (2022) suggested that sub-anesthetic doses of S-ketamine can delay the anesthetic recovery during laparoscopic cholecystectomy. Unlike Zhang et al.’s studies, there were no significant differences in PACU recovery and awakening time among the four groups. Interestingly, patients’ satisfaction in groups M-Sk and H-Sk was significantly higher than that in group C. On the one hand, this may be significantly related to the reduction of postoperative pain scores and the incidence of PONV. On the other hand, it may be related to the antidepressant effect of S-ketamine.

## 5 Limitations

Several limitations in this study should be noted. 1) The small sample size included in the trial may lead to an overestimation of the treatment effect. Subsequent studies with larger sample sizes and multi-center are required to verify the findings presented here in depth. 2) In this study, the protective effect of the intervention group on inflammation and immunosuppression is difficult to explain whether it is the effect of opioid-sparing or the direct effect of S-ketamine or both, which needs to be confirmed by a large number of *in vivo* and *in vitro* studies. 3) In addition, the immune-inflammatory protection effect of S-ketamine was more obvious in 0.5 or 0.75 mg/kg doses. Nevertheless, the optimal dose in our study remains undefined. Thus, more rigorous studies are needed to determine the optimal dose of S-ketamine administration, considering its anti-inflammatory effect, anti-immunosuppression efficacy, analgesic efficacy, and side effects.

## 6 Conclusion

Collectively, our study establishes that S-ketamine can be effectively used for perioperative analgesia, which also has anti-inflammatory and immune protection effects. It will greatly benefit the long-term prognosis of cancer patients and is worthy of clinical promotion. Moreover, we found that the effects of S-ketamine are related to the dose used, with significant differences observed between the 0.5 and 0.75 mg/kg doses of S-ketamine. These findings provide a new way to attenuate perioperative immunosuppression in patients with BC.

## Data Availability

The original contributions presented in the study are included in the article/Supplementary Material. Further inquiries can be directed to the corresponding author.

## Abbreviations

MRM, modified radical mastectomy; NK, natural killer cells; NLR, neutrophil-to-lymphocyte ratio; LMR, lymphocyte-to-monocyte ratio; PLR, platelets-to-lymphocyte ratio; SIRI, systemic inflammation response index; SII, systemic immune-inflammation index; FCM, flow cytometry.
